# Examination of degenerative and damaged glenohumeral joint tissue samples with calorimetric analyses

**DOI:** 10.3389/fcell.2025.1653732

**Published:** 2025-09-01

**Authors:** Dénes Lőrinczy, András Bata, László Bogdán, Árpád Pillér, Laszlo G. Nöt

**Affiliations:** ^1^ Department of Biophysics, Medical School, University of Pécs, Pécs, Hungary; ^2^ Department of Traumatology and Orthopaedics, Balassa János Teaching Hospital of Tolna County, Szekszárd, Hungary; ^3^ Center for Musculoskeletal Surgery, Trauma Unit, ‘Kaposi Mór’ Teaching Hospital of Somogy County, Kaposvár, Hungary; ^4^ Institute of Physiotherapy and Sport Science, Faculty of Health Sciences, University of Pécs, Pécs, Hungary

**Keywords:** differential scanning calorimetry, thermogravimetry, tendon, cartilage, cancellous bone, rotator cuff tear, trauma, reversed shoulder arthroplasty

## Abstract

Introduction: In recent decades, the operative treatment of degenerative changes of large joints has undergone significant changes. Similarly, new surgical methods have become available for the treatment of fractures affecting the joint surface. Despite the development of medical imaging procedures, it is still a challenge to accurately assess the state of the joints prior to surgery. Our workgroup has widely used differential scanning calorimetric analyses (DSC) for the evaluation of different human anatomical structures. Aims: The purpose of the study was to find a potential relationship between the calorimetric changes of human glenohumeral tissue samples and the results of medical imaging, macroscopical and histological examinations. Patients and methods: various type of tissue samples (rotator cuff tendon, hyaline cartilage and subchondral bone of the humeral head) was collected from patients underwent reversed shoulder arthroplasty with indication of rotator cuff tear arthropathy or Neer Type VI proximal humerus fractures. CT and MRI examinations were performed prior to surgery. Thermal parameters were detected using differential scanning calorimetry (DSC) and thermogravimetry (DTA/TG). Intraoperative macroscopic analysis and histology were done to confirm our findings. Results: we have demonstrated that thermal parameters and denaturation curves of different types of tissue samples followed the results of preoperative CT or MRI examinations, macroscopic findings and histological analyzes. These findings were supported by regression analysis, using score systems for radiological and other changes. Conclusion: our data suggest that the calorimetric analysis of intraoperatively collected tissue samples could be a reliable method for the clinical investigation of damaged (fractured) or degenerative joints.

## Introduction

In the recent decades, the number of arthroplasties has been significantly increased, due to the continuous development of prostheses and surgical techniques. Among arthroplasties, knee and hip prosthesis implants are performed most often. The third most frequent prosthesis implantation is the shoulder replacement (Wengler et al., 2014; Siddiqi et al., 2022). Because of the increasing average age, shoulder arthroplasties are performed with orthopedic indications due to degenerative changes and diseases of the shoulder joint more frequently (Farley et al., 2021; Lübbeke et al., 2017). From a traumatological point of view, an increasing number of shoulder arthroplasty is done worldwide, for the treatment comminuted, intraarticular 4-part proximal humerus fractures (Garrigues et al., 2012; Fitschen-Oestern et al., 2020; Collin et al., 2019).

The first generation of shoulder prostheses was developed by Professor Neer in the early 1950s (Jerosch and Heisel, 2003; Jaap, 2021). Since then, almost 70 years passed and the shoulder implants have undergone significant development. Nowadays total, reverse total, hemi and partial resurfacing prostheses are available for the treatment of shoulder joint arthritis, advanced rotator cuff (ROC) damage and intraarticular 4-part fractures associated with significant displacement.

There are several protocols available to support an orthopedic indication for shoulder replacement. In case of trauma indications (4-part fractures), the selection of the appropriate implant type could be challenging. The MRI examination, known as the ‘gold standard’ for assessing the condition of the shoulder joint, is not available in all cases. The use of MRI is limited by older metal implants, the presence of a pacemaker, or claustrophobia. Histological examinations can provide an accurate picture of the damage to a specific structure, but their preparation is time-consuming. In addition, most classifications (Hamada, Walch) based on non-MRI examinations (CT, US) indirectly assess the state of ROC and were basically developed for non-traumatic cases.

There is a need for novel examination methods that could be successfully used in the future as a supplement to medical imaging or histological examinations and could help determine the stage of shoulder joint degenerative diseases. Also, these methods could provide further details about the biochemical background of ROC, hyaline cartilage and subchondral bone damage.

It would be important especially for trauma patients, since in their cases, the preoperative functional tests cannot be performed to assess the condition of the ROC and the range of motion (ROM) of the shoulder joint.

Differential scanning calorimetry (DSC) has previously been used successfully in the research of degenerative and inflammatory diseases of the skeletal muscle system, to detect structural changes in collagen (Nöt et al., 2021; Bata et al., 2022a; Nöt et al., 2022). Furthermore, TG/DTA (thermogravimetry/differential thermal analysis) also provides reliable information about the thermal stability of the bone stock and its composition based on mass loss (Bata et al., 2022b; Lőrinczy et al., 2023). The purpose of the study was to find a potential relationship between the calorimetric changes of human glenohumeral tissue samples and the results of medical imaging, macroscopical and histological examinations.

## Patients and methods

### Surgical technique and sample collection

The operations were done under general anesthesia (ITN), and scalenus blockade was used as part of postoperative pain relief. All operations were performed by single, experienced shoulder surgeon at the same facility. The shoulder joint was exposed through deltopectoral approach. After opening the joint capsule, we identified the tuberculum maius humeri, on which three of the four members of the ROC insert (m. supra-et infraspinatus, m. teres minor). During the implantation of a hemiprosthesis, the integrity and proper fixation of the tubercles determines the subsequent function of the shoulder joint.

We cut through the m. biceps brachii long tendon at its point of origin. The humeral head was removed with an oscillating saw. After opening the medullary cavity, we cemented the stem of the appropriate size into the medullary cavity. In case of hemiprosthesis implantation, the glenoid fossa was left intact, the remaining tuberculum maius was attached to the stem with suture material. If there was an advanced degeneration of the shoulder joint and the function of the ROC was already missing, a reverse total shoulder prosthesis was implanted, during which a cup was also implanted. In this case, we also took cartilage tissue (5 × 5 × 2.5 mm size) and subchondral bone sample (5 × 15 mm bone cylinder) from the glenoid fossa–of the so-called glenohumeral contact area (GCA).

For the control group, samples were taken from young patients who suffered proximal humerus fracture (4-part fracture with significant dislocation) and the restoration of the joint surface was no longer possible. The criteria were that patients in the control group cannot have degenerative or inflammatory changes of the shoulder joint.

During the operations, samples were collected from the following tissues: humeral head cartilage, humeral head subchondral bone, rotator cuff (supraspinatus tendon attached to the tuberculum maius), biceps tendon intra-articular section, glenoidal cavity cartilage, subchondral bone of glenoidal cavity. To examine the ROC tendons, an approx. 5 × 5 × 10 mm tissue sample was usually taken from the supraspinatus tendon (an approx. 1.5 cm length of intraarticular part, corresponding to its critical zone). A 5 × 5 × 2.5 mm hyaline cartilage sample was taken from the surface of the humeral head (corresponding to the humerus - glenoid fossa contact surface). Another sample was collected from the subchondral bone of the humeral head and from the subchondral bone of the glenoid fossa with a 5 mm inner diameter cylinder drill.

The samples required for histological sections were placed in 4% formalin, and the tissue samples required for DSC and TG/DTA examinations were placed in cold physiological saline solution, then transported and stored deep-refrigerated until further processing.

We collected only tissues for our experiments that were supposed to be removed during the interventions, as part of the surgical procedure. All procedures followed were in accordance with the ethical standards of the responsible Regional and Institutional Research Ethics Committee and with the Helsinki Declaration of 1975, as revised in 2008.

### Medical imaging studies

The degenerative changes of the glenohumeral joint were analyzed using the different type of classifications: for ROC degeneration the Hamada classification, for degree of glenohumeral arthrosis the Walch classification, for degree of cartilage damage: Outerbridge classification was applied. Optimally, it would be important to perform both CT and MR examinations, since the different classifications are based on different imaging modalities.

For the preoperative planning of surgeries with orthopedic indications, we also have the option for implant planning software, using native X-ray images or CT-scans. CT scans were performed with a Siemens Somatom Perspective Dual 64/128, and MR examinations were done with a Siemens Magnetom Essenza 1.5 T type device. The radiological evaluation is part of the routine surgical planning and does not mean any extra burden for the patients.

### Differential scanning calorimetry

Differential scanning calorimetry was performed following our standard procedure and described in elsewhere (Nöt et al., 2022). Briefly, the stored samples were washed three times e and remained in a sterile buffer on 4 °C before starting the calorimetric examinations (max. half an hour). The measurements were made by a SETARAM Micro DSC-II calorimeter between 0 °C and 100 °C with a heating rate of 0.3 K/min. Conventional closed Hastelloy batch vessels (V = 1 mL) were used for the experiment to perform the thermal denaturation. Samples’ masses were between in mgs: 100–150. The sample buffer was used as a reference. The sample and reference vessels were equilibrated with a precision of ±0.1 mg. There was no need to do any correction from the point of view of heat capacity between sample and reference vessels. With the help of a two-point setting SETARAM peak integration calorimetric enthalpy was calculated from the area under the heat absorption curve then the other thermal parameters (denaturation or melting temperature (*T*_*m*_), range of denaturation (*ΔT*), half width of transition (*T*_*1/2*_) and calorimetry enthalpy (*ΔH*_*cal*_) data of samples) were compared. After ASCII conversion, the data was processed using the Origin 6.0 program.

### Thermogravimetry (TG) and differential thermal analysis (DTA)

Thermogravimetry (TG) and Differential thermal analysis (DTA) was also performed following our standard procedure and described in details elsewhere (Bata et al., 2022b; Lőrinczy et al., 2023). The thermoanalytical investigation of bone samples was performed by an SSC/5200 TG/DTA equipment made by SII Seiko Instruments (Japan). The sample holders were open aluminum crucibles with a diameter of 5.2 mm and a depth of 5 mm. The investigated temperature range was from ambient up to 550 °C (aluminum sample holders). The applied heating rate was between 10–40 K/min (in 10 K steps). Measurements were performed under an inert nitrogen gas with a flow rate of 100 mL/min. The detected signals were DTA („heat flow”), TG (mass loss in %) and DTG („speed” of mass loss) curves.

During the thermogravimetric analyses, the percentage change in mass of the tested material were registered as a function of the temperature increase. The obtained thermogram provides information about sample’s thermal stability. DTG (derivative thermogravimetry) is registered at the same time as the TG curve and represents the derivative TG curve, calculated from the data points of the TG curve.

In case of differential thermal analysis (DTA), the tested sample (with an inert substance) is heated at a standard rate, while the temperature difference between the two substances is measured and registered as a function of heating. These data reflect to the enthalpy change in the examined sample.

### Statistical analyzes

The statistical analyses of DSC data were performed using GraphPad Prism 6 (GraphPad, San Diego, CA) and SPSS 2 6.0 (SPSS, Chicago, IL) software, where differences of p < 0.05 were considered statistically significant. For the evaluation of thermogravimetric tests, the correlation analysis and the creation of graphs were done using the MS Excel software.

## Results

### Patient demographics

Since 2019, a total of 51 patients have received hemi or reverse shoulder replacement in our department. By gender, the male:female ratio was 13:38. At the time of surgery, the average age (mean ± SEM, min – max., years) was 69 ± 1.1 (51–86) years. We performed shoulder arthroplasty in a total of 36 cases with a traumatological indication. In this group, all patients had 4-part or Neer Type VI (AO/ASIF 11 C3.2 - C3.3 [5a]) proximal humerus fractures, the fracture line extending to the collum anatomicum as well. Regarding the mechanism of injury, low-energy injuries (n = 31) were predominantly found. Therefore, the existing osteoporosis may be a significant risk factor for the comminuted 4-part proximal humerus fractures. High-energy injuries (n = 5) - such as “falling from a height” or “being run over by a vehicle” - occurred only in a small number of cases. Epilepsy (n = 1), drunkenness (n = 1) and general deterioration caused by COVID-19 infection (n = 1) were occasionally included as other factors in the cause of injuries.

In a total of 15 cases, the prosthesis implantation was performed with orthopedic indications. In these cases, reverse shoulder arthroplasty was done with cemented (n = 4) or cementless (n = 11) stem. In all patients with orthopedic indications, advanced rotator cuff tear arthropathy (RCA) was the indication for surgery, often accompanied by a history of degenerative and/or autoimmune disease.

### Functional outcome of shoulder replacement with orthopaedic and trauma indications

**FIGURE 1:**
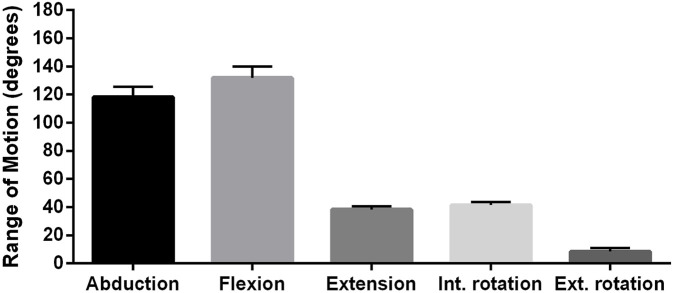
Range of motion (ROM) of trauma patients 12 months after reversed shoulder replacement operated.

In cases of surgery performed due to comminuted proximal humerus fractures, the preoperative range of motion cannot be examined because of the pain and loss of function caused by the fracture. Therefore, in trauma cases, the ‘before and after’ comparison of shoulder function was not available. Figure 1 indicates the range of motion 12 months after surgery.

**FIGURE 2:**
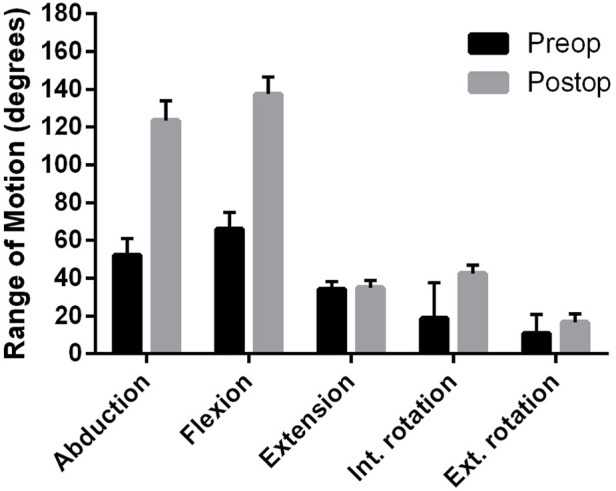
Comparison of range of motion (ROM) of orthopaedic patients before and 12 months after reversed shoulder replacement (data are mean ± SEM).

In orthopedic cases, however, it is possible to compare the range of motion before and after surgery, thereby obtaining functional data to evaluate the success of the shoulder replacement. Figure 2 clearly shows that the abduction and anteflexion movements of the shoulder joint have significantly improved compared to the pre-operative function.

**FIGURE 3:**
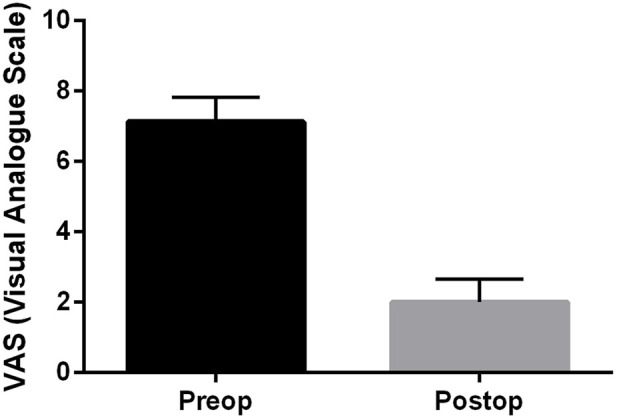
Comparison of range of motion (ROM) of orthopaedic and trauma patients 12 months after reversed shoulder replacement (data are mean ± SEM).

A comparison of the range of motion of orthopedic and trauma cases indicated that there was no significant difference between the two patient groups in terms of the achieved range of motion (Figure 3).

**FIGURE 4:**
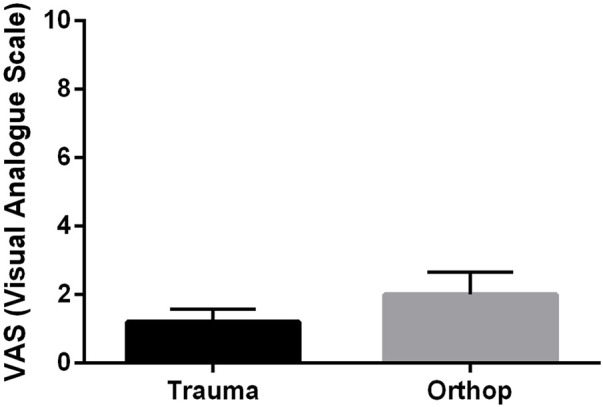
Comparison of pain score (VAS) of orthopaedic patients before and 12 months after reversed shoulder replacement (data are mean ± SEM).

In our studies, we examined the degree of pain, for which we used a numerical scale analogous to the visual analogue scale (VAS). This is a one-dimensional pain scale, which practically consists of a horizontal axis on which the patient must mark the degree of pain. One end point is the inscription “no pain at all” (=0), and the other is the inscription “unbearably painful” (=10). The average of the values before and after orthopedic surgeries (12 months) is shown in Figure 4, which proves that the intervention significantly reduced the patients’ pain.

**FIGURE 5:**
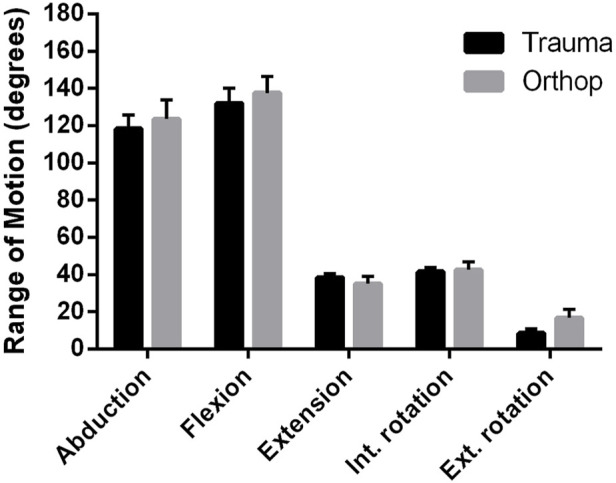
Comparison of pain score (VAS) of orthopaedic and trauma patients 12 months after reversed shoulder replacement (data are mean ± SEM).

VAS scores were compared between orthopedic and traumatology patients after 12 months following shoulder replacement. surgery. The results showed that there was no significant difference in pain between the two groups after surgery (Figure 5).

Intra- and postoperative complications were also evaluated. Intraoperative periprosthetic fractures occurred in 3.9% of the procedures performed. Wound healing disorders and superficial septic complications were also observed in the same proportion. Deep septic complications were not detected during the follow-up period. Aseptic loosening of the baseplate occurred in 2% of patients.

### Medical imaging and macroscopic analyzes

In different stages of osteoarthritis, the glenoid and the humeral head’s position in relation to the glenoid are evaluated by the Walch classification. Grade A1 in sample F2 shows a well-centered humeral head with mild erosions, while grade D in sample D2 shows very severe arthritis with anterior humeral head dislocation (Table 1).

**TABLE 1:** Macroscopic and radiological evaluation of different tissue samples of patients orthopaedic shoulder diseases (D) or acute comminuted proximal humerus fractures (F**)** Table published here with permission of Journal of Thermal Analysis and Calorimetry (Lőrinczy et al., 2023).

Sample	Macroscopic score			Medical imaging
	ROC	Cartilage	Bone	OA score
Degenerative				
D1	4	4	2–3	6
D2	5	5	4	5
Fracture				
F1	3	3	4	2
F2	4	2	2	4
F3	3	2–3	1	0

Grade 4a and grade 5 cuff tear arthritis were identified in orthopaedic samples based on the Hamada classification. In contrast, trauma sample F3 had a grade 4b (glenohumeral arthritis with subacromial acetabulization) degree of damage.

Additionally, the B1 orthopaedic sample had a grade 2 (blister-like swelling/fraying of articular cartilage spreading to surface) osteochondral lesion, according to the MRI-based modified Outerbridge classification.

Notably, all of these classifications were largely developed for the assessment degenerative (RCA) shoulders; as a result, they have limited relevance for trauma shoulders. In the meantime, it was evident from the intraoperative macroscopic assessment and from osteoarthritis score that the orthopedic indication specimens had progressed tissue degeneration and osteoarthritis. Histological analysis of the control samples verified the presence of healthy tissue structure.

### Calorimetric analyzes

#### Shape of DSC curves of rotator cuff (ROC) samples

**FIGURE 6:**
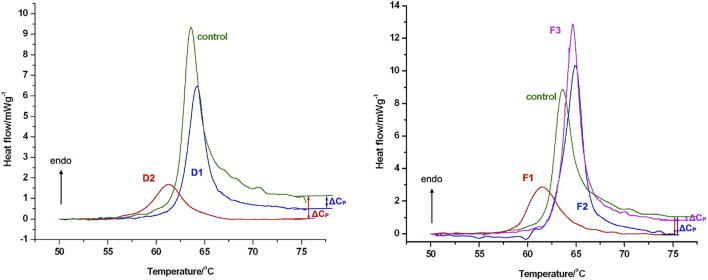
The thermal denaturation scans of rotator cuff (ROC) samples of patients with orthopaedic shoulder diseases or acute comminuted proximal humerus fractures. Samples were run in technical triplicates and the heat flow curves show the averages of three measurements. Data were normalized to wet sample mass. Upward arrow indicates endotherm reaction. Figure published here with permission of Journal of Thermal Analysis and Calorimetry (Lőrinczy et al., 2023).

In comparison to the control, the curves’ shape amply illustrates the impact of moderate tendon damage on sample D1’s tendon structure and the developed tendon degeneration in sample D2’s. In contrast to the morphology of the control (healthy) sample, the denaturation curves of the degenerative (orthopaedic) samples are flattened and expanded (Figure 6).

#### Thermal parameters of rotator cuff (ROC) samples

When compared to controls, the calorimetric enthalpy significantly decreased, indicating pathologic changes in the collagen structure of the degenerative tendons taken from orthopaedic patients. In contrast to the control, the degenerative samples had a smaller temperature range of denaturation (Table 2). After denaturation, the baseline may shift, indicating a higher heat capacity and a more compact (dense) structure due to decreased tendon elasticity.

**TABLE 2:** Thermal parameters of rotator cuff samples of patients with orthopaedic shoulder diseases (D) or acute comminuted proximal humerus fractures (F) ***(*** *ΔT* - temperature range of denaturation, T_1/2_ - half width at the maximal heat flow, T_m_–the peak temperature of the denaturation an ***d*** *ΔH*_*cal*_–*calorimetric enthalpy*). Each sample was run in triplicates, data are the average of three measurements**.** Table published here with permission of Journal of Thermal Analysis and Calorimetry (Lőrinczy et al., 2023).

Sample	Thermodynamic parameters			
	*ΔT/°C*	*T*_*1/2*_ */°C*	*T*_*m*_ */°C*	*ΔH*_*cal*_ */Jg* ^*-1*^
Degenerative				
Control	18.60	2.02	63.55	5.01
D1	11.71	1.83	64.24	3.14
D2	15.49	2.16	61.45	1.83
Fracture				
Control	18.60	2.02	63.55	5.01
F1	13.23	2.76	61.60	2.69
F2	12.56	1.93	64.93	5.33
F3	12.15	1.83	64.60	5.67

The format of the scans and the thermal characteristics of the moderately weakened tendons (F1) differed significantly from the control. In contrast to the control and other fracture samples (F2, F3), the contour of the denaturation curve was expanded and flattened in the case of fracture sample F1. In contrast to the fracture samples, the control sample’s curve has a sharper slope.

The thermal enthalpy in the less injured F2 and F3 samples was moderately increased, however the F1 fracture sample with degenerative macroscopic symptoms exhibited a significant drop compared to the intact control. The temperature range of denaturation was reduced in all fracture samples. When compared to the control, the F1 sample had a larger half width at the maximum heat flow and a lower peak temperature of denaturation. The alterations in these parameters revealed an opposite pattern: T_1/2_ decreased while T_m_ increased in compared to the control.

#### Shape of DSC curves of cartilage samples

**FIGURE 7:**
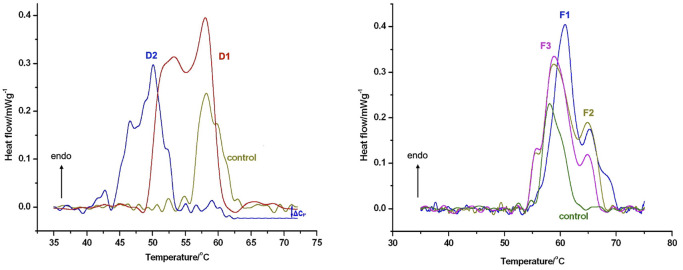
The thermal denaturation scans of hyaline cartilage samples of patients with orthopaedic shoulder diseases or acute comminuted proximal humerus fractures. Samples were run in technical triplicates and the heat flow curves show the averages of three measurements. Data were normalized to wet sample mass. Upward arrow indicates endotherm reaction. Figure published here with permission of Journal of Thermal Analysis and Calorimetry (Lőrinczy et al., 2023).

Our primary conclusion regarding sample origin and illness outcome is that the degenerative samples (D) have a much larger denaturation temperature range and half-width of the heat flow curves than the control (C) and fracture (F) samples. Only in the upward portion of the curves may an additional peak be seen in the thermal denaturation curves of degenerative samples (Figure 7).

Furthermore, the denaturation curves of fracture samples exhibit thinner and less noticeable additional peaks throughout both the climbing and falling phases. The exact reason for those additional peaks is outside the purview of our current investigation because we do not yet have the biochemical tools to distinguish between the different cartilage components.

#### Thermal parameters of cartilage samples

The thermal enthalpy of both degenerative samples differed markedly from the control. Interestingly, thermal enthalpy resulted in either a minor rise in fracture samples F2 and F3, or a significant decrease in sample F1 (Table 3). Meanwhile, the melting temperature of the D2 sample with the most severe degenerative alterations decreased when compared to the control.

**TABLE 3:** Thermal parameters of cartilage samples of patients with orthopaedic shoulder diseases (D) or acute comminuted proximal humerus fractures (F) ***(*** *ΔT* - temperature range of denaturation, T_1/2_ - half width at the maximal heat flow, T_m_–the peak temperature of the denaturation an ***d*** *ΔH*_*cal*_–*calorimetric enthalpy*). Samples were run in triplicates and data were normalized on the wet mass of the samples**.** Table published here with permission of Journal of Thermal Analysis and Calorimetry (Lőrinczy et al., 2023).

Sample	Thermodynamic parameters			
	*ΔT/°C*	*T*_*1/2*_ */°C*	*T*_*m*_ */°C*	*ΔH*_*cal*_ */Jg* ^*-1*^
Degenerative				
Control	19.0	7.9	56.5	0.62
D1	27.1	17.6	56.0	1.84
D2	20.0	10.5	50.0	1.04
Fracture				
Control	19.0	7.9	56.5	0.62
F1	17.1	4.3	61.1	0.41
F2	15.0	8.9	58.9	1.07
F3	14.6	5.0	58.9	1.09

Each sample in the fracture group showed a greater Tm than the control or orthoapedic samples. In comparison to the control, all samples of orthopaedic cartilages displayed an increase in the denaturation temperature range. On the other hand, the ∆T decreased in all trauma samples.

The considerable decrease in D2 melting temperatures (Tm) demonstrated that degenerative diseases cause greater structural change than traumatic exposure. These results show the thermal impact of injured cartilage’s lower cooperativity between structural domains.

#### DTA/TG analyzes of subchondral cancellous bone samples

**FIGURE 8:**
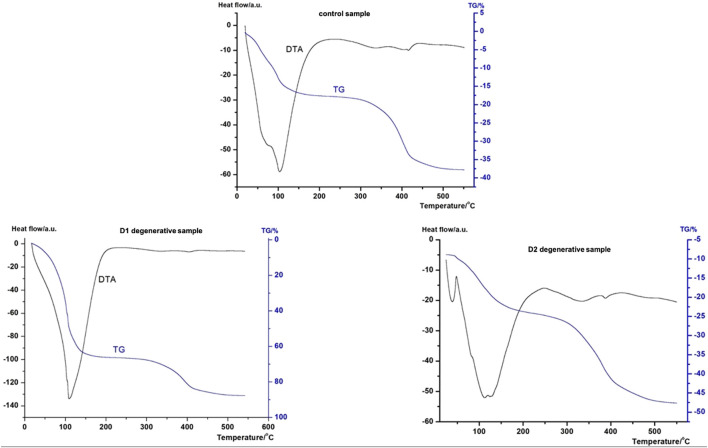
TG/DTA scans of cancellous bone samples of patients with orthopaedic shoulder diseases. Samples were run in technical triplicates and the heat flow curves show the averages of three measurements. Data were normalized to dry sample mass. Figure published here with permission of Journal of Thermal Analysis and Calorimetry (Lőrinczy et al., 2023).

**FIGURE 9:**
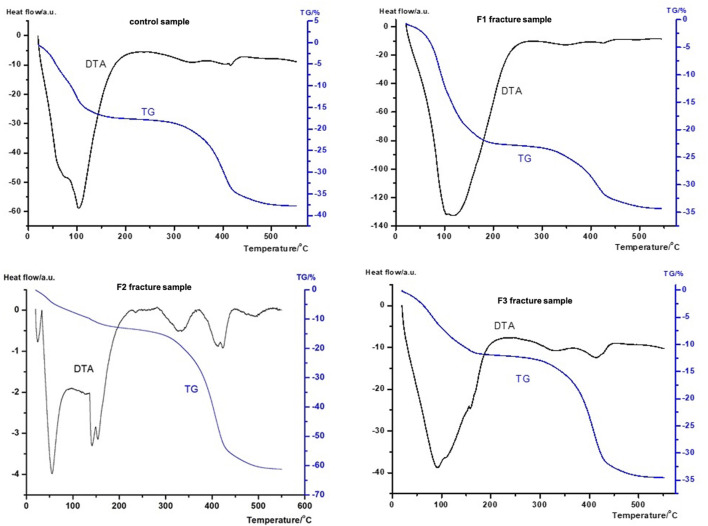
TG/DTA scans of cancellous bone samples of patients with acute comminuted proximal humerus fractures. Samples were run in technical triplicates and the heat flow curves show the averages of three measurements. Data were normalized to dry sample mass. Figure published here with permission of Journal of Thermal Analysis and Calorimetry (Lőrinczy et al., 2023).

The DTA result for the control sample revealed a total mass loss of approximately forty percent in the temperature range under investigation, along with four separate thermal transitions. (Figures 8, 9). During the upward phase, the DTG curves (data not shown) showed a pattern comparable to the DTA’s morphologies; there are two distinct peak temperatures, 1.5 mg/min and 2.1 mg/min. On the other hand, only the DTG demonstrated distinct peaks at 402 °C at 2.7 mg/min. Additionally, when compared to the D2 and control runs, the DTA curve of D1 was noticeably narrower.

Furthermore, the D1 degenerative sample exhibited twice the thermal effect as the control specimen. Lower temperature peaks reached 50 °C and 107 °C, with a four-fold increase in endothermic impact compared to the control setting. At that point, the higher transitions remained at approximately the same temperature (333 °C and 404 °C). The three graphs showed a twice larger final mass loss (Table 4).

**TABLE 4:** Thermodynamic parameters (DTA measurements), mass loss (Δm) and DTG parameters of subchondral cancellous bone samples of patients with orthopaedic shoulder diseases or acute comminuted proximal humerus fractures *(T*_*low*_–thermal effect at lower temperature, *T*_*high*_ - thermal effect at higher temperature, *ΔH*_*cal*_–calorimetric enthalpy). Sample were run in triplicates and thermal enthalpies were calculated based on the total denaturation range**.** Table published here with permission of Journal of Thermal Analysis and Calorimetry (Lőrinczy et al., 2023).

	DTA thermodynamic parameters			
Sample	T_low_/°C	ΔH_cal_/Jg^-1^	T_high_/°C	ΔH_cal_/Jg^-1^
Degenerative				
Control	60.1–103.2	682	335.7–407.2	42.2
D1	46.5–108.2	8504.7	333.8–404.7	153.2
D2	38–118	4849.4	332.6–387.3	394
Fracture				
Control	60.1–103.2	682	335.7–407.2	42.2
F1	45.7–117.5	2190	348.1–423	36.5
F2	56–141–153	329.5	335.1–410.6	21.4
F3	91.1–157.6	564.2	331–413.6	49
mass loss/%				
Degenerative				
Control	7.5–15		20–30	
D1	30		45	
D2	17.5		40	
Fracture				
Control	25		50	
F1	35		60	
F2	15		45	
F3	15		50	

	DTG parameters (mg/min)			
	T_low_/°C	DTG	T_high_/°C	ΔH_cal_/Jg^-1^
Degenerative				
Control	60.1–98.9	1.5–2.1	402.3	2.7
D1	107.4	4.61	392.3	1.3
D2	120	1.4	381.1	2.3
Fracture				
Control	60.1–98.9	1.5–2.1	402.3	2.7
F1	100.4	4.4	360–414.3	1.2–2.7
F2	47.8–143	0.2–0.4	338–408	0.3–1.2
F3	84.8	1.1	407.7	3.3

In contrast to the D1 degenerative sample, the D2 sample exhibited features and heat denaturation that were comparable to the control. The two denaturation peaks, for example, separated more clearly in the lower range, yielding the same calorimetric enthalpy. Three peaks were seen in the higher denaturation range, compared to the control sample’s two peaks. Denaturation at 333 °C had a greater enthalpy contribution than the control at 335 °C and a significantly greater one than the second at 388 °C. About 40% was the overall mass lost.

There is a noticeable difference in the denaturation process between the fracture group and the degenerative samples (Figure 9). Consequently, it also suggests a distinct variation in the cancellous bone structure. The F1 showed changes in temperature from 50 (inflection) to 120 °C, along with twice the control’s calorimetric enthalpy.

Denaturation at higher temperatures shifted to 348 °C and 423 °C, with approximately the same enthalpies. The DTG value rose (4 mg/min) at 100.4 °C when compared to the control. In the higher range, DTG values were comparable to the control.

The F2 sample was the most different from the control or any fracture or degenerative sample. A single transition at around 50 °C, a well-separable double denaturation at about 150 °C, and the others at 340, 420, and 500 °C were found in a clear pattern. It is noteworthy, that the overall calorimetric enthalpy in the 20 °C–220 °C range was significantly lower than in the control. About 45% was the overall mass lost.

Thermal changes at 90, 110, and 150 °C can be identified in the F3 sample. The enthalpy contribution of these three peaks falls within the control range of 20 °C–210 °C. At higher temperatures, the enthalpies are similar to the control. The DTG values were 85 °C, 1.12 mg/min, and 408 °C, 3.3 mg/min. The total mass loss was 50 percent.

#### Comparison of the degree of tissue damage, degeneration and thermal parameters

To determine whether the thermal parameters and other markers of degenerative processes, like macroscopic scores or radiological evaluations, are related, regression analyses have been conducted. The osteoarthritis score and denaturation temperature range of the cartilage samples showed a “moderately strong” correlation (*R* ^2^ = 0.74, p = 0.06, trend to statistical significance). Additionally, a similar pattern was shown when the osteoarthritis score was plotted as a predictor and the half width at the maximum heat flow was shown as a dependent variable (*R* ^2^ = 0.76, p = 0.05, trend to statistical significance).

The degree of arthritis (osteoarthritis score) and the calorimetric enthalpy were shown to be causally related at the lower temperature peaks when the results of the radiological evaluation and the calorimetric measurements were compared (*R* ^2^ = 0.61, p = 0.067, trend toward significant). Note that the association would be significantly greater (*R* ^2^ = 0.92) if one outliner (sample F2) were removed.

## Discussion

Although protocols and guidelines have advanced recently, research is still required to examine the biochemical basis of rotator cuff degeneration, hyaline cartilage, and subchondral bone damage (Smith et al., 2017; Launonen et al., 2019; Wilcox et al., 2005). These investigations may give the surgeon additional information regarding the anticipated severity of arthritis, especially when an MRI scan is not available.

The pathophysiology of rotator cuff injuries has been the subject of numerous recent research, but few of them have used differential scanning calorimetry (DSC) to assess thermodynamic parameters. For instance, Szabó et al. used an experimental animal model of rabbit rotator cuff ruptures to demonstrate clear distinctions between normal and damaged tendons (Szabó et al., 2006).

Utilizing DSC, they found that the pathologic biceps tendons had a higher thermal enthalpy, lower melting temperatures, and a loss of thermal cooperation of the constituents. The increased amount of collagen and secondary linkages in the collagen fibers were found as a possible explanation (Szabó et al., 2007). Additionally, it was shown that samples from freshly injured lower extremity tendons had a much higher melting temperature, since damaged tendons had a reduced quantity of bound water (Wiegand et al., 2013).

Age, excessive use, and recurrent microtraumas have all been implicated in the development of rotator cuff tears and the consequential shoulder arthropathies (Trębacz et al., 2018). Bognár et al. found notable, age-related alterations in the long biceps head’s thermal denaturation capabilities using tendons from human cadavers. The age-dependent thermal enthalpy in their study peaked at 51 years old and displayed a broad range (Bognár et al., 2010). It should be mentioned that whilst we used *in vivo* samples for all of our investigations, those studies used healthy human tendons that were taken from cadavers.

Based on the DSC curve morphologies and thermodynamic characteristics of samples from different origins, we showed that structural alterations in human hyaline cartilage caused by degenerative conditions are more significant than those seen in case of 4-part or Neer Type IV fractures. The structural damage identified in trauma patients may be the result of a chronic injury or the trauma itself. When comparing the histopathologic alterations of the humeral head between osteoarthritis (OA) and cuff tear arthropathy (CTA), it was discovered that the cartilage layer was thicker in CTA than in OA (Toma et al., 2019).

In a previous study, Csotye at el. have analyzed glenohumeral arthritis patients’ joint surfaces. Their study concluded that DSC is a useful technique for distinguishing between samples of normal hyaline cartilage and degenerated cartilage, despite the fact that their sample collection and analysis methods differed from our technique (Csotye et al., 2009).

The human hyaline cartilage is classified into three zones: superficial, transitional, and deep, each with its unique function and consistency. Surprisingly, we noticed that the thermal denaturation scans of certain samples displayed ‘transitions,’ which could be caused by an interaction between one of the components of the distinct zones.

Another series of trials looked at how thermal properties were affected by the amount of time that had passed since the femoral neck fracture. They discovered variations in thermal enthalpy and melting temperature, which supported the theory that the human hyaline cartilage sustains structural alterations in the event of an inadequate blood supply (Naumov et al., 2012).

Damage to hyaline cartilage and a decrease in the mineral content of subchondral bone are hallmarks of osteoarthritis, a degenerative joint disease. Although the bone is hypomineralized and of worse quality than in a healthy cancellous structure, osteoarthritis causes an increase in the volume of bone and the number of trabeculae (Henrotin et al., 2012; Bettica et al., 2002). We regularly observed a comparatively higher mass loss when comparing the more impacted orthopedic sample (D1) to the control. In contrast, the trauma samples displayed mass loss that was similar to that of the control.

The subchondral bone plate, located directly beneath the cartilage, and the deeper trabecular bone are the two anatomical components that make up the subchondral structure (Li et al., 2013; Gol et al., 2010). Four different thermal transitions were found in the control sample, according to our thermal analyses. The endotherm effect increased fourfold in the D1 orthopaedic sample due to a shift in the lower temperature transition peaks. Higher summits, meanwhile, stayed in the same temperature range. The other orthopaedic sample’s (D2) thermal characteristics were comparable to the control’s.

One possible explanation is that the multiple peaks represent distinct subchondral bone layers. The deeper layers that are less impacted are represented by the higher, thermodynamically more stable transitions, whereas the lower peaks correspond to the more responsive transition zone or subchondral plate (Suri and Walsh, 2012).

Interestingly, the thermal characteristics of the F2 trauma sample differed greatly from those of the other samples. For instance, three transitions were detected at 340 °C, 420 °C, and 500 °C, one transition at about 50 °C, and a well-separable double denaturation close to 150 °C. The enthalpy contribution was higher than in the control in all three denaturation phases. Additionally, at 60%, the final mass loss was the largest. Either more severe acute microstructural damage or rapid avascular necrosis caused by the acute trauma could be responsible for the different trend of the curves.

Using regression analysis on samples of hyaline cartilage and subchondral cancellous bone, we have shown a reasonably important correlation between temperature parameters and osteoarthritis scores. It's interesting to note that there was no statistically significant relationship between the results of thermal data and radiological scores in the case of the rotator cuff samples.

One explanation could be that the osteoarthritis score is determined by the glenohumeral joint’s radiological morphology, which describes the joint space, bone consistency, etc. The extent of rotator cuff tendon injury cannot be determined by CT scans or standard radiography. Only indirectly could the radiological morphology of the joint be used to estimate potential degenerative changes of the rotator cuff tendon.

It's not always evident how samples with orthopedic or trauma origins differ from one another. Orthopedic patients may experience AVN symptoms due to prior trauma, and trauma patients may have symptoms of pre-existing osteoarthritis. We most likely saw the “outlining” trauma pattern as a result of both acute trauma and the heightened impact of degenerative changes.

As a result, we believe that data demonstrating changes in the status of ROC, hyaline cartilage, and subchondral bone between trauma and orthopedic patients might be helpful in surgical planning and therapy procedures.

### Potential limitations

A potential limitation of the study is that radiological evaluation was only possible in a limited number of cases. This is because the available classifications were developed for shoulder joints affected by RCA degeneration and not for the evaluation of multi-fragmentary fractures. In addition, MRI is not always available due to certain factors.

Another limitation of the study is that due to the relatively small number of samples, only mean values ​​were calculated without further statistical evaluations. However, the marked differences in the denaturation curves of healthy and pathological samples and the excellent reproducibility of the measurements (temperature: ±0.2 °C, enthalpy <0.05 J/g) support our conclusion that thermodynamic analysis is a reliable tool for the evaluation of degenerative tissue samples.

During the surgeries, cylindrical bone samples of 5 × 15 mm (diameter x height) were taken, which included both layers of the subchondral bone - cortical and cancellous. Therefore, the thermal analysis was performed on the entire cylinder and data from both layers were collected during the same experiment. As a result, it cannot be excluded that the larger sclerotic part of the subchondral cysts influences the measurement.

It should be noted that the p-value of our data only showed a trend of significance (p < 0.1). However, based on the *R* ^2^ values, we believe that in a future study with a larger sample size, we would find a strong correlation between radiological and thermal data.

## Conclusion

Our data suggest that the calorimetric analysis of intraoperatively collected tissue samples could be a reliable method for the clinical investigation of damaged (fractured) or degenerative joints.
